# Clinical Assessment of Hypothyroid Patients Using the Zulewski Score and the Correlation Between Thyroid and Lipid Parameters: A Tertiary Care Centre-Based Study From Central India

**DOI:** 10.7759/cureus.88288

**Published:** 2025-07-19

**Authors:** Vaibhav Yadav, Rajesh Kumar Jha, Akash Pawar, Abhishek Singhai, Ritika Agarwal, Mahadev Meena, Gaurav Runwal, Bhupendra Chouhan, Aman Yadav, Monica Razdan

**Affiliations:** 1 Department of General Medicine, Mahatama Gandhi Memorial Medical College, Indore, IND; 2 Department of General Medicine, Sri Aurobindo Institute of Medical Sciences and Research Centre, Indore, IND; 3 Department of General Medicine, All India Institute of Medical Sciences, Bhopal, Bhopal, IND; 4 Department of Anaesthesiology, Gajra Raja Medical College, Gwalior, IND; 5 Department of General Medicine, Mahatma Gandhi Memorial Medical College, Indore, IND

**Keywords:** hypothyroidism, lipid parameters, thyroid disorders, type 2 diabetes mellitus, zulewski score

## Abstract

Introduction: Clinical scores based on signs and symptoms have been used in thyroidology to identify individuals at high risk for hypothyroidism. The current study observes the signs and symptoms of patients with hypothyroidism who are positive for hypothyroidism according to the Zulewski clinical score, and determines associations between thyroid and lipid profiles.

Methods: The current prospective cohort study was conducted among newly diagnosed hypothyroid patients aged 18-65 years with a Zulewski score > 5 (indicative of hypothyroidism) and biochemically diagnosed as hypothyroid. Demographics, thyroid, and lipid profiles of the patients were recorded and analysed. A P < 0.05 was considered to be statistically significant.

Results: The current study included 100 patients with hypothyroidism (M:F = 1:1.94), with an average age of 40.9 ± 12.1 years. Slow movement and coarse skin were the most common signs among men (67.6%) and women (68.2%), respectively. Weight gain was the most common symptom (M: 91.2%; F: 87.9%). The TSH levels of patients significantly increased with higher Zulewski scores. A deranged lipid profile was observed in 86% of patients. A positive correlation was observed between total cholesterol and TSH (r = 0.275; P = 0.006), and between total cholesterol and T4 (r = -0.205; P = 0.041). A negative correlation was observed between T3 and cholesterol (r = -0.263; P = 0.008), and between T3 and triglycerides (r = -0.263; P = 0.008).

Conclusion: The current study highlights the reliability of the Zulewski score for early diagnosis and risk assessment in Indian patients with hypothyroidism. It could also serve as an indirect clinical measure to assess deranged lipid parameters and identify the risk of atherosclerosis among hypothyroid patients.

## Introduction

Hypothyroidism is associated with significant morbidity and mortality. It affects around one-tenth of the Indian population (11%) and is twice as common as its Western counterparts [1-4]. Hypothyroidism is also associated with an elevated risk of developing cardiovascular disease [2,3] and metabolic disorders [4].

The endocrine abnormalities in patients with hypothyroidism increase the risk of dyslipidaemia (odds ratio: 3.56; 95% CI: 1.29-9.84; p = 0.01) [5]. The deranged lipid profile leads to progressive lipid accumulation, contributing to plaque formation in the arteries, which may increase the risk of cardiovascular disease-related morbidity and mortality [6,7].

For early detection of hypothyroidism, clinical scores based on signs and symptoms have been developed and can be used to identify individuals at high risk [8,9]. Zulewski et al. reassessed classical signs and symptoms of hypothyroidism in the light of modern laboratory tests [8]. The Zulewski score is a clinical feature-based, simple bedside assessment tool used to detect hypothyroidism and related risk stratification, such as lipid abnormalities. Among elderly Indian patients, studies have observed a sensitivity of 80-100%, with approximately 50% specificity for the Zulewski score in identifying hypothyroidism [10,11]. However, the application of the Zulewski score in the younger Indian population for identifying the increasing risk of hypothyroidism is lacking [9]. The use of such clinical scores may aid in the early diagnosis of hypothyroidism in this population [9].

Therefore, this India-based study aims to evaluate the correlation between the Zulewski score and lipid abnormalities in patients with hypothyroidism.

## Materials and methods

The current prospective cohort observational study was conducted among patients attending the outpatient or inpatient department at a medical college in central India.

Inclusion criteria

Inclusion criteria included individuals within the age group of 18-65 years; patients who were clinically diagnosed with hypothyroidism and satisfied Zulewski’s clinical score of >5 (indicative of hypothyroidism); and patients with TSH >10 μIU/dL and T4 <5.4 μg/dL.

Exclusion criteria

Exclusion criteria included patients who were clinically diagnosed as hypothyroid according to the Zulewski score but had thyroid function tests within the normal range; patients taking medications affecting thyroid function, such as levothyroxine, glucocorticoids, and antiepileptics; patients taking medications affecting the lipid profile, such as lipid-lowering agents (statins, fibrates); and patients with diseases or conditions known to alter TSH secretion, such as chronic renal failure, liver disease, neoplasms, pregnancy, or immunocompromised status.

The study was conducted in accordance with the Declaration of Helsinki, 1964. The patients or their legal guardians were informed about the procedures, benefits, and future prospects. Following this, voluntary written informed consent was obtained. The study obtained Institutional Ethics Committee approval prior to initiation (Reference number: SAIMS/IEC/2016/69; approved on November 25, 2016). Written permission was obtained from the original authors to use the score in this study.

Data collection

Demographic details of the hypothyroid patients, including age, sex, and BMI, were collected. Subsequently, the Zulewski score was estimated by a trained clinician using a standardised checklist during the initial clinical evaluation (Table 1) [8,12].

**Table 1:** Estimation of Zulewski score Minimum score: 0; Maximum score: 12 Calculation of Zulewski score based on the presence or absence of certain symptoms [13]. Reproduced with permission from Zulewski et al. [8], ©1997 by Oxford University Press.

Symptoms	Details	Present	Absent
Diminished sweating	Sweating in a warm room or on a hot summer day	1	0
Hoarseness	Speaking voice singing voice	1	0
Paranesthesia	Subjective sensation	1	0
Dry skin	Dryness of skin, noticed spontaneously, requiring treatment	1	0
Constipation	Bowel habit, use of laxatives	1	0
Impairment of hearing	Progressive impairment of hearing	1	0
Weight increase	Recorded weight increase tightness of clothes	1	0
Physical signs			
Slow movements	Observe the patient removing his clothes	1	0
Delayed ankle reflex	Observe the relaxation of the reflex	1	0
Coarse skin	Examine hands, forearms, and elbows for roughness and thickening of skin	1	0
Periorbital puffiness	This should obscure the curve of the malar bone	1	0
Cold skin	Compare the temperature of the hands with the examiners	1	0

Among those with a Zulewski score >5 (indicative of hypothyroidism), thyroid function tests (T3, T4, TSH) were conducted. Patients who were found to be biochemically overt hypothyroid, i.e., TSH >10 mIU/dL and T4 <5.4 μg/dL, were included. On the following day, the fasting lipid profile of these patients was collected. Dyslipidemia was defined as per the criteria of the National Cholesterol Education Program [13]. There were no missing data in the dataset, and all enrolled patients had complete thyroid and lipid profiles.

Statistical analysis

The patient details were stored in Excel (Microsoft Corporation, Redmond, Washington) and analysed using IBM SPSS Statistics for Windows, Version 26 (Released 2019; IBM Corp., Armonk, New York). Categorical variables were expressed as absolute numbers (N) and frequency (%), and continuous variables as mean ± SD and/or median [IQR]. Categorical variables were compared using the chi-square test, and continuous variables using parametric or non-parametric Student’s t-tests, as appropriate, based on the normality of data. Correlation between two groups was assessed using Karl Pearson’s correlation coefficient. Associations between continuous and discrete variables were calculated using a one-way ANOVA test, and pairwise comparisons were conducted using the Student-Newman-Keuls post hoc test. A p-value < 0.05 was considered statistically significant.

## Results

The current study included 100 patients with hypothyroidism, with an average age of 40.9 ± 12.1 years. Individuals between the ages of 30-49 years comprised 62% of the total population. About 66% of the study population were women. The average BMI of the study population was 26.2 ± 4.8 kg/m², with the majority (40%) having category I obesity (25.0-29.9 kg/m²). Table 2 compares the demographics, thyroid profile, and lipid profile for gender-based differences.

A deranged lipid profile was observed in 86% of patients. High total cholesterol levels were present in 29%, high LDL levels in 36%, low HDL levels in 58%, and high triglyceride levels in 52% of the study population (Table 2). A higher percentage of men had total cholesterol under control compared to women (47.0% vs 30.3%; P = 0.0225). The average Zulewski score of the study population was 7.3 ± 1.1 and was not significantly different between the genders.

**Table 2:** Comparison of demographics and clinical parameters between the genders # t-statistic. ## Chi-square statistic. BMI: body mass distribution; TC: total cholesterol; HDL: high density lipoprotein; LDL: low density lipoprotein.

Variables	Total population	Male	Female	Test statistic	p-value
N	100	34	66		
Age (years)	40.95 ± 12.1	40.3 ± 15.5	41.0 ± 11.9	0.251^#^	0.803
BMI (kg/m^2^)	26.2 ± 4.8	26.2 ± 4.8	26.2 ± 4.8	0.000^#^	0.999
BMI distribution					
<18.0 (underweight)	1 (1.0%)	0 (0%)	1 (1.5%)	7.966^##^	0.158
18.0–22.9 (Normal)	23 (23.0%)	6 (17.6%)	17 (25.7%)		
23.0–24.9 (Overweight)	16 (16.0%)	4 (11.8%)	12 (18.2%)		
25.0–29.9 (Obese I)	40 (40.0%)	14 (41.2%)	26 (39.4%)		
30.0–34.9 (Obese II)	17 (17.0%)	10 (29.4%)	7 (10.6%)		
>35.0 (Obese III)	3 (3.0%)	0 (0%)	3 (4.5%)		
Serum TSH (μIU/mL)	Values missing	42.5 ± 25.2	43.0 ± 27.3	-0.500^#^	0.929
Serum T3 (ng/dl)	0.9 ± 0.3	0.9 ± 0.3	0.9 ± 0.3	0.000^#^	0.999
Serum T4 (mg/dl)	4.0 ± 1.4	4.0 ± 1.4	4.0 ± 1.4	0.000^#^	0.999
TC (mg/dL)	213.5 ± 48.1	213.2 ± 48.2	213.5 ± 48.1	-0.300^#^	0.977
TC (mg/dL) distribution					
<200 (Desirable)	34 (34.0%)	16 (47.0%)	20 (30.3%)	7.588^##^	0.022
200–239 (Borderline)	37 (37.0%)	4 (11.8%)	25 (37.9%)		
>240 (High)	29 (29.0%)	14 (41.2%)	21 (31.8%)		
LDL cholesterol (mg/dL)	143.7 ± 40.7	144.4 ± 41.3	143.7 ± 40.7	0.700^#^	0.936
LDL cholesterol (mg/dL) distribution					
<100 (Optimal)	17 (17.0%)	5 (14.7%)	12 (18.2%)	2.120^##^	0.713
100–129 (Near optimal)	17 (17.0%)	8 (23.5%)	13 (19.7%)		
130–159 (Borderline high)	30 (30.0%)	9 (26.5%)	21 (31.8%)		
160–189 (High)	25 (25.0%)	10 (29.4%)	15 (22.7%)		
≥190 (Very high)	11 (11.0%)	2 (5.9%)	9 (13.6%)		
HDL cholesterol (mg/dL)	37.5 ± 10.9	37.1 ± 10.2	37.5 ± 10.9	-0.400^#^	0.859
Distribution of HDL cholesterol levels (mg/dL)					
<40 (Low)	58 (58.0%)	21 (61.8%)	37 (56.1%)	2.187^##^	0.335
40–59 (Normal range)	38 (38.0%)	13 (28.2%)	25 (37.9%)		
≥60 (High)	4 (4.0%)	0 (0%)	4 (6.0%)		
Triglyceride (mg/dL)	226.6 ± 99.6	228.0 ± 90.5	226.6 ± 99.4	1.400^#^	0.945
Distribution based on triglyceride (mg/dL)					
<150 (Desirable)	16 (16.0%)	6 (17.6%)	10 (15.2%)	0.423^##^	0.935
150–199 (Borderline high)	32 (32.0%)	10 (29.4%)	22 (33.3%)		
200–499 (High)	50 (50.0%)	17 (50%)	33 (50%)		
≥500 (Very high)	2 (2.0%)	1 (3%)	1 (1.5%)		
Zulewski score	7.3 ± 1.1	7.3 ± 1.2	7.3 ± 1.1	0.000^#^	0.999
Zulewski score, median (IQR)	7.0 (6.0, 8.0)	7.0 (6.0, 8.2)	7.0 (6.0, 8.0)		

Distribution of signs and symptoms of hypothyroidism according to the Zulewski score

**Figure 1:**
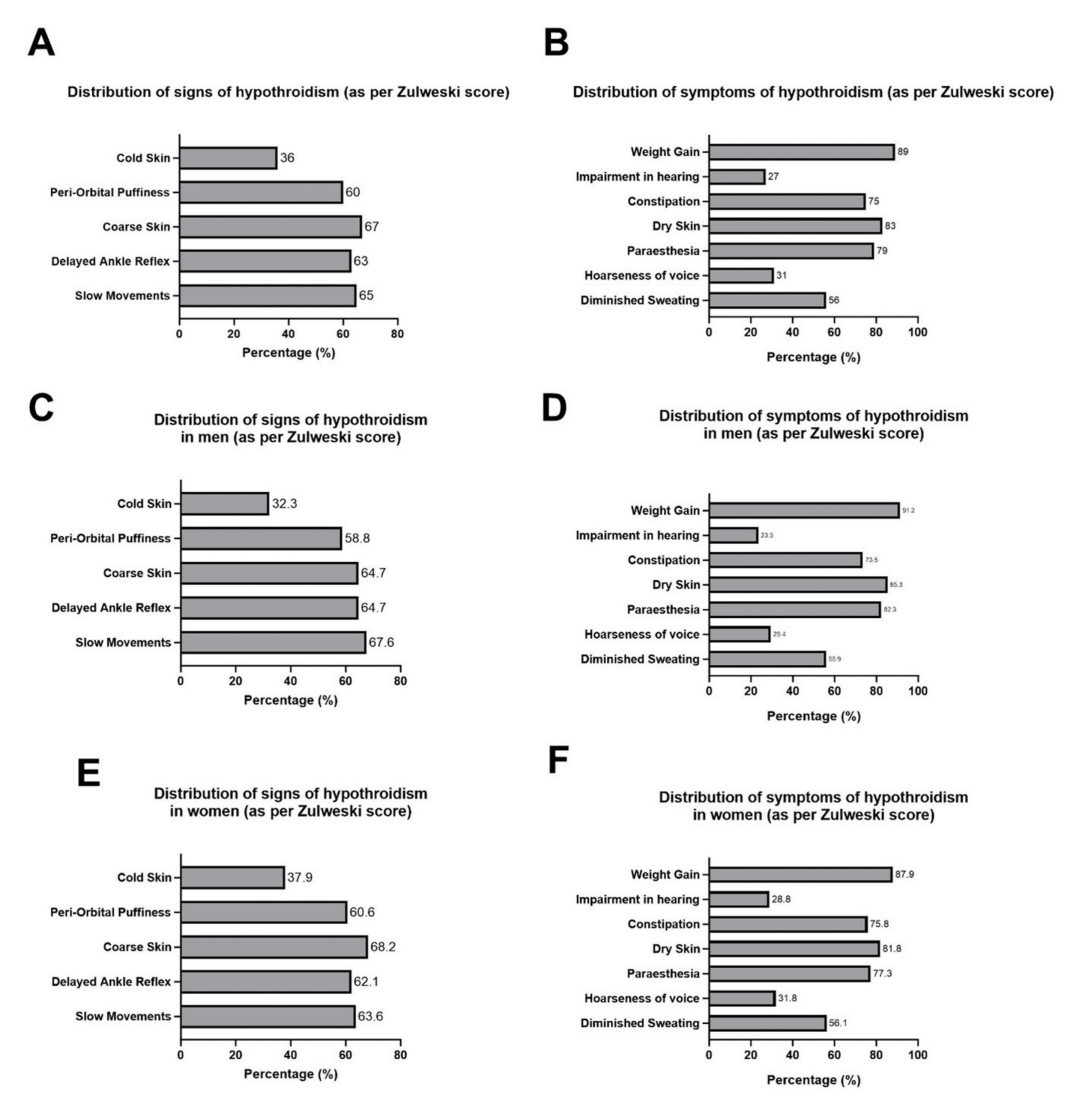
Distribution of signs and symptoms of hypothyroidism according to Zulewski score A, C, and E represent the distribution of prevalent signs of hypothyroidism according to the Zulewski score in the total population, men, and women, respectively. B, D, and F represent the distribution of prevalent symptoms of hypothyroidism according to the Zulewski score in the total population (N = 100), men (N = 34), and women (N = 66), respectively.

Coarse skin was the most common sign of hypothyroidism (observed in 67% of participants) according to the Zulewski score (Figure 1A). Weight gain was the most common symptom (observed in 89%) among the study participants (Figure 1B). Regarding gender-based differences, slow movements were the most common sign among men (67.6%) (Figure 1C), while coarse skin was the most common sign among women (68.2%) (Figure 1D). Weight gain was the most common symptom in both men (91.2%) and women (87.9%) (Figure 1E,F).

**Figure 2:**
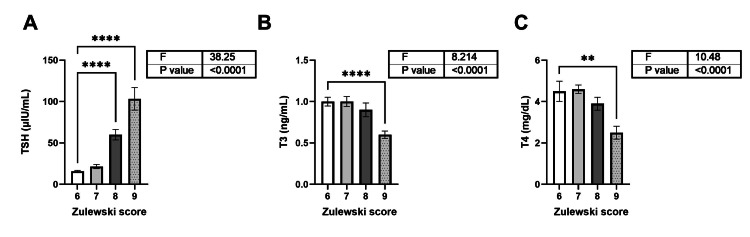
Comparison of mean TSH, T3, and T4 levels according to the Zulewski score. A, B, and C represent the comparison of thyroid hormones TSH, T3, and T4, respectively, in a population with increasing Zulewski scores. The bars represent the mean, and the error bars represent the SEM. The statistical test used was one-way ANOVA with multiple pairwise comparisons. ** and **** represent P < 0.01 and P < 0.001, respectively. TSH: Thyroid-stimulating hormone; T3: triiodothyronine; T4: thyroxine.

The mean TSH, T3, and T4 levels were calculated for each Zulewski score. TSH levels in patients with Zulewski scores of 8 and 9 were significantly higher than those with a score of 6 (Figure 2A). Patients with a Zulewski score of 9 also had significantly lower T3 and T4 levels compared to those with a score of 6 (Figures 2B,C).

The mean differences in TSH, T3, and T4 levels were estimated for populations with different Zulewski scores. The difference in mean TSH levels was highest between populations with Zulewski scores of 9 and 6 (MD = 87.45; P < 0.001) (Table 3). The difference in mean T3 levels was highest between populations with Zulewski scores of 9 and 6 (MD = -0.4608; P < 0.001). The difference in mean T4 levels was highest between populations with Zulewski scores of 9 and 7 (MD = -2.154; P < 0.001).

**Figure 3:**
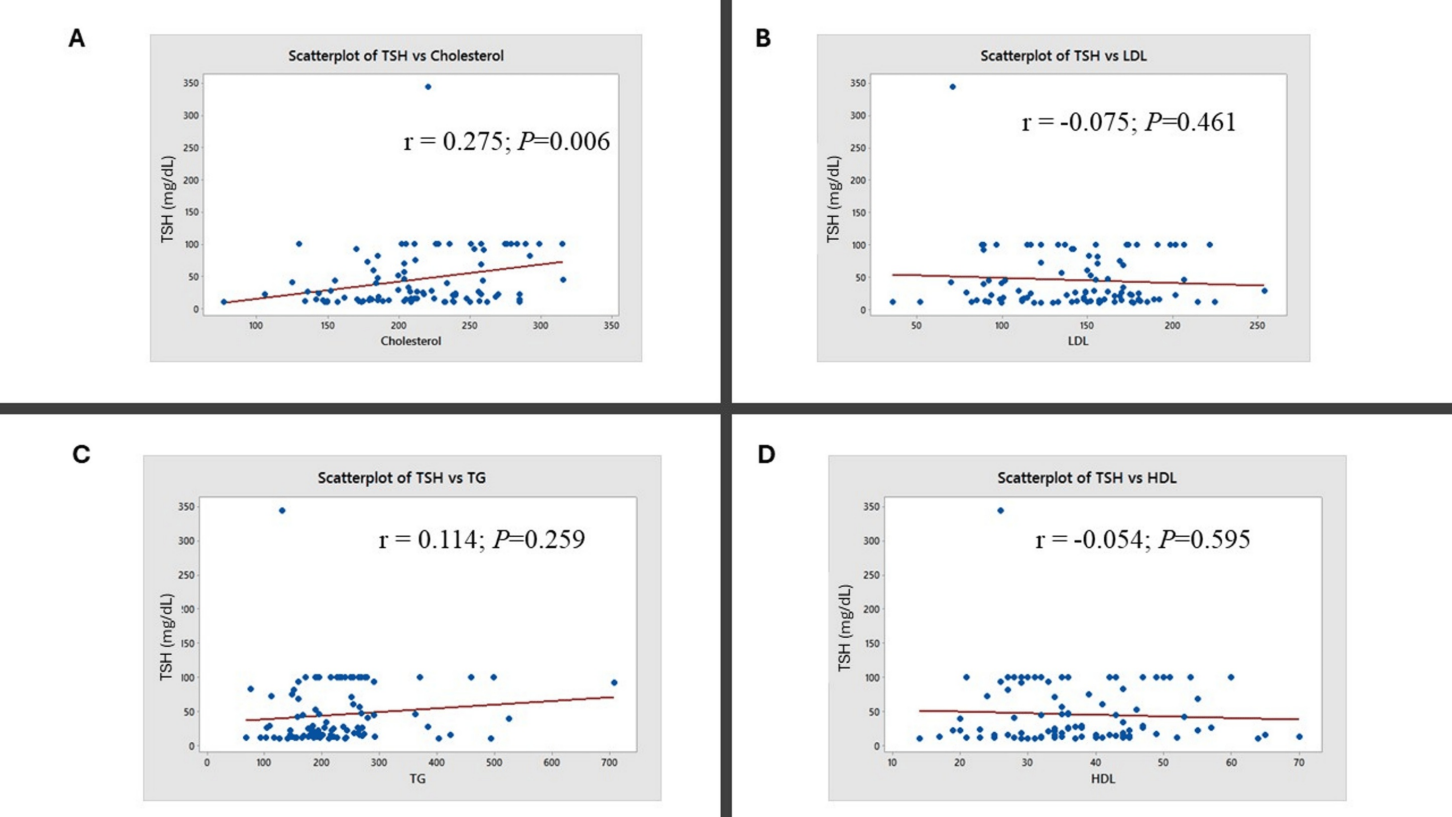
Correlation of TSH with lipid parameters The figure represents the correlation (red line) between TSH and (A) total cholesterol, (B) low-density lipoprotein (LDL), (C) triglycerides (TG), and (D) high-density lipoprotein (HDL). Karl Pearson’s correlation test was used. The correlation coefficient (r) and the corresponding P-values are indicated.

**Figure 4:**
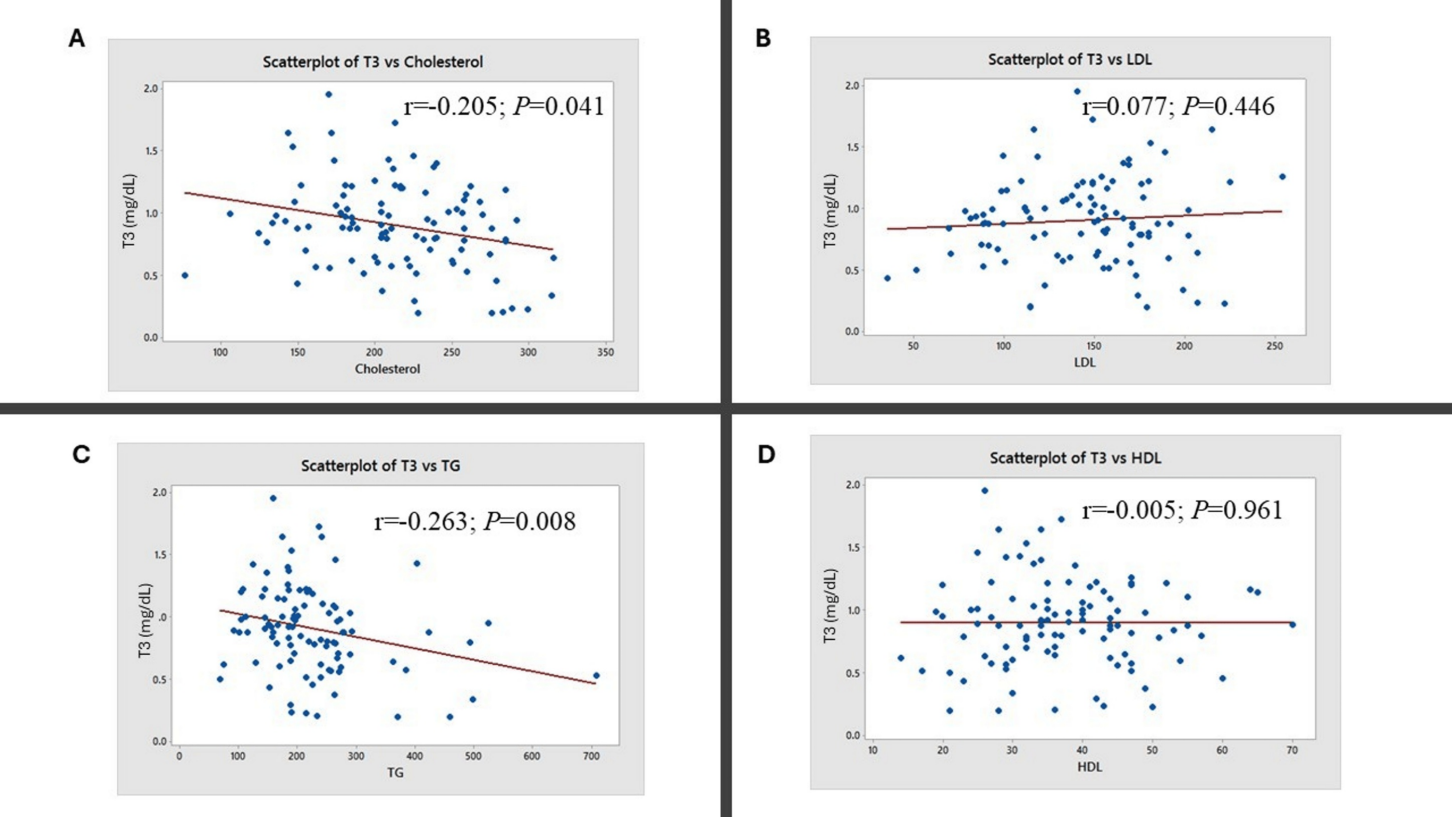
Correlation of T3 with lipid parameters The figure represents the correlation (red line) between T3 and (A) total cholesterol, (B) low-density lipoprotein (LDL), (C) triglycerides (TG), and (D) high-density lipoprotein (HDL). Karl Pearson’s correlation test was used. The correlation coefficient (r) and the corresponding P-values are indicated.

**Figure 5:**
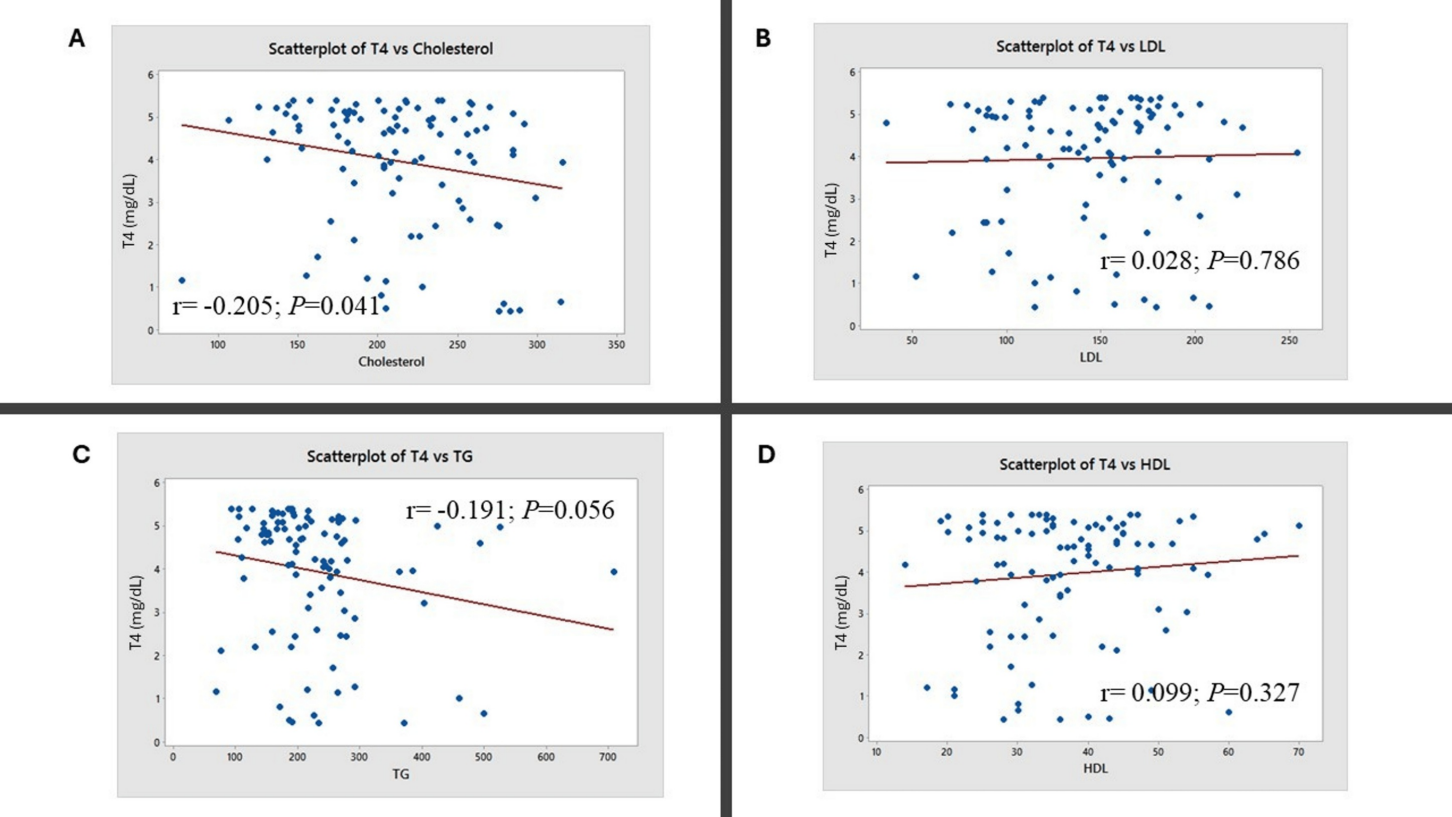
Correlation of T4 with lipid parameters The figure represents the correlation between thyroxine (T4) and (A) total cholesterol, (B) low-density lipoprotein (LDL), (C) triglycerides (TG), and (D) high-density lipoprotein (HDL). Karl Pearson’s correlation test was used. The correlation coefficient (r) and the corresponding P-values are indicated.

Further correlation analysis was conducted between thyroid and lipid parameters (Figures 3-5). A positive correlation was observed between total cholesterol and TSH levels (r = 0.275; P = 0.006; Figure 3A). A negative correlation was observed between T3 and cholesterol levels (r = -0.263; P = 0.008; Figure 4A) and between T3 and triglyceride levels (r = -0.263; P = 0.008; Figure 4B). A negative correlation was observed between total cholesterol and T4 levels (r = -0.205; P = 0.041; Figure 5A).

## Discussion

The current study highlights the symptomatic profile of Indian hypothyroid patients based on the Zulewski clinical score and investigates the correlation between thyroid function and lipid parameters. A total of 100 patients were enrolled using a consecutive sampling approach, of whom 66% were women. Previous Indian studies suggest a threefold higher prevalence of hypothyroidism among women compared to men [1]. Thus, screening for hypothyroidism, especially in women, is essential for early diagnosis and risk stratification.

An increase in Zulewski score was associated with a significant increase in TSH levels and a corresponding decrease in T3 and T4 levels (Figure 2). These findings are consistent with prior Indian research demonstrating elevated TSH levels in association with higher Zulewski scores [9]. Among the clinical features of hypothyroidism, slow movements were most common among men (67.6%), whereas coarse skin was most frequently observed in women (68.2%). Hypothyroid myopathy affects approximately 30-80% of patients [14] and is thought to result from disrupted glycogen and oxidative metabolism in the actin-myosin complex [15]. A previous study also noted a higher prevalence of coarse skin among women with hypothyroidism compared to men (86.7% (women) vs. 68.6% (men)) [9], suggesting gender-based differences in clinical presentation.

Weight gain was the most commonly reported symptom in both men (91.2%) and women (87.9%), consistent with the observation that 40% of the study population had Class II obesity. Reduced thermogenesis and metabolic rate are considered major contributors to weight gain and obesity in hypothyroid patients [16]. This metabolic disturbance is also responsible for the high prevalence of dyslipidemia (~90-96%) in this population [5,17]. Hypothyroidism has been shown to increase the risk of dyslipidemia by 3.56 times (95% CI: 1.29-9.84; p = 0.01) in obese individuals [5].

In this study, 86% of participants had a deranged lipid profile. Elevated total cholesterol was noted in 29%, elevated LDL in 36%, reduced HDL in 58%, and elevated triglycerides in 52% of patients (Table 2). A study by Khatri et al. (2021) involving 58 overt hypothyroid patients also reported similar findings, with 55-69% showing lipid abnormalities [18]. Correlation analysis in our study revealed a significant positive relationship between TSH and total cholesterol levels (Figure 3A), along with a significant negative correlation between T3 and both total cholesterol (Figure 4A) and triglyceride levels (Figure 4C). The correlation analysis showed statistically significant but modest associations between thyroid and lipid parameters. A positive correlation between TSH and total cholesterol suggests that declining thyroid function may worsen lipid profiles, increasing cardiovascular risk. Negative correlations between T3 and both cholesterol and triglycerides support the role of thyroid hormones in lipid metabolism. The weaker inverse correlation between T4 and cholesterol aligns with this trend. While clinically relevant, these findings should be viewed as supportive rather than conclusive due to the modest strength of the correlations. These observations are supported by the findings of Tarboush et al. (2023), who demonstrated a positive correlation between TSH and cholesterol and a negative correlation between T4 and total cholesterol among 324 patients with subclinical and overt hypothyroidism [15]. 

Thyroid hormones play a vital role in lipid metabolism and mobilization. TSH influences cholesterol homeostasis by interacting with receptors on hepatocytes [19] and adipocytes [20]. LDL cholesterol biosynthesis is regulated by HMG-CoA reductase, which is controlled by thyroid hormones. Reduced thyroid hormone levels in hypothyroidism lead to decreased HMG-CoA reductase activity, promoting intracellular LDL-C accumulation and downregulation of LDL receptors, resulting in reduced LDL clearance [6,7,11]. In our study, a majority of patients exhibited elevated total cholesterol and LDL-C levels, which aligns with the underlying mechanism of reduced HMG-CoA reductase activity and decreased LDL receptor expression in hypothyroidism. Although HDL-C levels were not the primary focus, the observed lipid abnormalities are consistent with impaired lipid clearance described in hypothyroid states.

In addition, hypothyroidism impairs HDL-C synthesis by reducing apolipoprotein A1 (ApoA1) production, which is crucial for cholesterol efflux from peripheral tissues [7]. Although some studies report elevated HDL-C levels in severe hypothyroidism [21,22], this may not translate into improved cardiovascular health, as HDL-C efflux capacity remains impaired due to reduced ApoA1 levels [23]. Furthermore, reduced activity of cholesteryl ester transfer protein (CETP), which facilitates HDL remodeling, contributes to impaired HDL clearance [11]. Therefore, even patients with normal or elevated HDL-C may be at risk for cardiovascular events due to reduced HDL functionality [23].

This single-center study has limitations, including a modest sample size that reduces statistical power and limits the generalizability of findings. The observational design does not establish causality, and potential confounding factors such as diet, comorbidities, and medication use were not controlled.

Our findings align with previous research demonstrating a correlation between hypothyroidism and dyslipidemia, as well as the clinical utility of the Zulewski score. Given its simplicity, non-invasiveness, and low cost, the Zulewski score may be useful in resource-limited settings as a screening tool to identify individuals at risk and guide further testing.

## Conclusions

In conclusion, the Zulewski score can assist in identifying and assessing the risk of hypothyroidism in clinical settings, particularly where access to laboratory tests is limited. It may also serve as an indicator of potential lipid abnormalities and cardiovascular risk. This study was limited by its single-center design, small sample size, and lack of adjustment for confounding factors, which may affect the generalizability and interpretation of the findings. Larger, multi-center studies with appropriate adjustments are needed to confirm these results and evaluate the routine use of the Zulewski score in hypothyroidism screening.
